# Supplementary material for: Development and evaluation of a food frequency questionnaire for use among young children
Source: PLoS One. 2020 Mar 25;15(3):e0230669. doi: 10.1371/journal.pone.0230669 (PMC7094848; doi:10.1371/journal.pone.0230669)
Supplement: S1 Fig — (DOCX) [file pone.0230669.s001.docx]

Number of days between FFQ and 24-hour recall : <7 days

24 hr recall 3

Weekend day

24 hr recall 1

Weekday

FFQ

24 hr recall 2

Weekday

S1 Fig: Assessment period between FFQ and three 24 hour recalls in the InFANT study.
