# Supplementary material for: Development and evaluation of a food frequency questionnaire for use among young children
Source: PLoS One. 2020 Mar 25;15(3):e0230669. doi: 10.1371/journal.pone.0230669 (PMC7094848; doi:10.1371/journal.pone.0230669)
Supplement: S1 Table — (DOCX) [file pone.0230669.s002.docx]

S1 Table. Comparison of children included and excluded from the analysis

| **Variable** | **Age 1.5 years** | | | **Age 3.5 years** | | | **Age 5.0 years** | | |
| --- | --- | --- | --- | --- | --- | --- | --- | --- | --- |
|  | Included | Excluded | p-value* | Included | Excluded | p-value* | Included | Excluded | p-value* |
| Sample size, n | 231 | 261 |  | 172 | 189* |  | 187 | 175* |  |
| Sex, n (%) male | 128 (55.4%) | 136 (52.1%) | 0.46 | 84 (48.8%) | 108 (57.1%) | 0.11 | 95 (50.8%) | 96 (54.9%) | 0.44 |
| Age of child, mean (SE) | 1.5 (0.01) | 1.8 (0.01)^ | P<0..001 | 3.6 (0.01) | 3.7 (0.02) | P<0..001 | 5.0 (0.01) | 5.1 (0.01)^ | P<0..001 |
| Body Mass Index z-score, mean (SE) | 0.84 (0.06) | 0.82 (0.07)^ | 0.12 | 0.70 (0.06) | 0.55 (0.07)^ | 0.12 | 0.61 (0.07) | 0.44 (0.08) | 0.10 |
| Maternal education, n (%)* |  |  | 0.67 |  |  | 0.72 |  |  | 0.42 |
| Low | 51 (22.1%) | 52 (19.9%) |  | 30 (17.4%) | 38 (20.1%) |  | 34 (18.2%) | 33 (18.9%) |  |
| Intermediate | 52 (22.5%) | 67 (25.7%) |  | 36 (20.9%) | 42 (22.2%) |  | 37 (19.8%) | 44 (25.1%) |  |
| High | 128 (55.4%) | 142 (54.4%) |  | 106 (61.6%) | 109 (57.7%) |  | 116 (62.0%) | 98 (56.0%) |  |

*Chi-squared test a t-test were used where appropriate

**Does not include participants who did not participate in data collection at that time point

^Age of child at 1.5yrs, n=247; BMI at 1.5yrs, n=231; BMI at 3.5ys, n=182; Age of child at 5.0yrs, n=169
