# Supplementary material for: Development and evaluation of a food frequency questionnaire for use among young children
Source: PLoS One. 2020 Mar 25;15(3):e0230669. doi: 10.1371/journal.pone.0230669 (PMC7094848; doi:10.1371/journal.pone.0230669)
Supplement: S2 Table — (DOCX) [file pone.0230669.s003.docx]

S2 Table: Categorization of 26 food groups used in comparison of FFQ with the 24-hour recalls

| Categories | Categories from FFQ items |
| --- | --- |
| Plain Water | Water |
| Milk | Full fat, Skim milk, Reduced fat milk, Soy milk, Flavoured milk, Other milk, infant formula |
| All other beverages | Fruit Juice, Diet soft drinks, Soft drinks, Other beverages |
| Cheese | Cheese |
| Yoghurt | Yoghurt |
| Cream, Ice-cream, Custard | Cream, Ice cream |
| Non-white bread | Wholegrain bread, Rye bread, Wholemeal bread |
| White bread | White bread, High Fibre white bread, Other breads |
| Breakfast Cereal | High Fibre breakfast cereals, Other breakfast cereals |
| Rice, Pasta and Other cereal products | Rice, pasta, other cereal products |
| Red meat and meat dishes | Beef and lamb, beef and lamb dishes, pork, pork dishes |
| Poultry and poultry dishes | Chicken, Crumbed chicken, Chicken dishes, Other poultry dishes |
| Seafood and seafood dishes | Fish dishes, Crumbed fish, Fish and Seafood |
| Processed meat | Ham, Sausages, Other processed meat |
| Eggs | Chicken eggs, Other eggs |
| Fruit | Apples and pears, Grapes, Kiwi fruit, Oranges, Peaches, Pineapples, Rockmelon, Strawberries, Watermelon, Other fruit, Fruit with added sugar, Dried fruit |
| Vegetables (No potatoes) | Broccoli, Capsicum, Carrots, Corn, Cauliflower, Cucumber, Mushrooms, Onions, Peas, Pumpkin, Tomatoes, Zucchini, Other vegetables |
| Potato | White potato, Other potato varieties |
| Hot chips | Hot chips |
| Takeaway style foods | Hamburgers, pizza, savoury pies |
| Sweet snacks | Cakes, Chocolate, Sweet biscuits |
| Savoury Snacks | Potato crisps, Savoury biscuits |
| Crispbreads/Crackers | Crackers and crispbreads |
| Nuts and Seeds | Nuts and seeds |
| Butter/Margarine | Butter, Margarine |
| Sugars, jams, honey | Sugar |
