# Supplementary material for: Development and evaluation of a food frequency questionnaire for use among young children
Source: PLoS One. 2020 Mar 25;15(3):e0230669. doi: 10.1371/journal.pone.0230669 (PMC7094848; doi:10.1371/journal.pone.0230669)
Supplement: S3 Table — (DOCX) [file pone.0230669.s004.docx]

S3 Table 3. Comparison of energy-adjusted food intakes as estimated by the FFQ and the 24-hour recalls

| **Food item** | **Age 1.5 years (n=231)** | | **Age 3.5 years(n=172)** | | **Age 5.0 years (n=187)** | |
| --- | --- | --- | --- | --- | --- | --- |
|  | **FFQ** | **24-Hour recall** | **FFQ** | **24-Hour recall** | **FFQ** | **24-Hour recall** |
| Water | 812 (771-1047) | 302 (194-475) | 914 (526-1163) | 469 (293-676) | 1125(1125-1500) | 539 (353-731) |
| Milk | 438 (320-493) | 410 (307-511) | 341 (210-468) | 282 (150-375) | 265 (170-457) | 239 (142-324) |
| All other beverages | 2.1 (0-9.8) | 0.2 (0-0.8) | 24.0 (8.5-95.2) | 31.1(1.7-138.7) | 43.6 (15.3-141.1) | 46.8 (5.1-117.4) |
| Cheese | 17.4 (11.5-22.2) | 11.5 (3.7-19.0) | 16.7(10.1-20.5) | 16.1 (5.5-25.1) | 16.8 (9.0-20.1) | 18.3 (9.3-28.9) |
| Yoghurt | 83.4(53.3-102.3) | 53.1(19.5-89.0) | 59.7(33.3-96.5) | 47.5(15.2-90.4) | 55.0 (23.1-94.7) | 45.0 (14.9-88.3) |
| Cream/ ice-cream/custards | 3.5 (0.5-8.0) | 1.7 (0-7.5) | 7.1(3.3-12.2) | 2.2 (0-12.7) | 6.3 (3.6-12.3) | 5.3 (0.3-15.2) |
| Non-white bread | 38.8 (16.8-66.9) | 20.6 (5.3-36.2) | 57.6(24.9-95.2) | 33.9(10.1-54.8) | 61.4 (17.9-109.1) | 25.8 (5.8-61.9) |
| White bread | 6.1 (0-20.9) | 9.8 (1.1-29.1) | 14.8 (0.6-39.3) | 16.7 (2.3-38.6) | 15.7 (0-45.9) | 27.6 (4.5-55.1) |
| Breakfast cereal | 24.8 (18.0-29.8) | 25.3(15.1-40.6) | 27.0(14.2-32.0) | 25.4(10.9-49.7) | 24.9 (12.9-31.0) | 28.7 (10.7-50.8) |
| Rice/pasta/other cereals | 57.7 (37.0-82.9) | 41.6(18.5-73.0) | 64.3(38.0-86.2) | 43.3(21.4-79.8) | 71.5 (42.6-107.5) | 54.1(28.1-101.6) |
| Red meat | 35.6 (22.7-52.9) | 15.8 (4.5-32.2) | 37.9(23.5-61.4) | 14.6 (1.0-36.9) | 46.0 (27.7-64.3) | 16.5 (1.9-43.3) |
| Poultry | 15.8 (8.8-24.1) | 4.1 (0.1-17.7) | 21.9(12.4-33.8) | 9.0 (0.7-23.8) | 22.3 (12.9-35.5) | 13.3 (0.1-39.9) |
| Seafood | 13.5 (7.0-21.0) | 2.1 (0-12.2) | 18.1 (9.8-30.3) | 0.5 (0-18.2) | 14.1 (8.6-24.2) | 2.6 (0.1-25.2) |
| Processed meat | 2.1 (0.6-4.2) | 1.7 (0-10.1) | 6.0 (2.5-11.2) | 14.2 (4.2-31.4) | 13.0 (7.8-21.9) | 11.4 (1.9-19.6) |
| Eggs | 4.2 (1.8-5.7) | 0.9 (0-8.0) | 4.5 (2.1-14.0) | 0 (0-14.7) | 6.6 (3.2-16.6) | 0.8 (0-15.3) |
| Fruit | 187.4(132.6-259.2) | 145.3(98.5-206.7) | 268 (210-355) | 191 (117-264) | 363 (263-464) | 198 (135-279) |
| Vegetables (no potatoes) | 87.8 (60.4-121.7) | 30.8 (14.5-71.4) | 89.0(58.2-128.1) | 52.3(27.5-90.8) | 102(74.9-149) | 71.2 (34.8-119) |
| Potato | 9.1 (4.3-26.2) | 1.5 (0-13.3) | 9.3 (4.1-26.6) | 5.5 (0.5-27.3) | 10.3(4.6-30.7) | 3.9 (0-30.8) |
| Hot chips | 3.8 (0.2-4.6) | 0.1 (0-0.3) | 5.7 (1.9-8.9) | 2.5 (0-14.7) | 5.9 (3.5-10.2) | 0.7 (0-7.2) |
| Takeaway style foods | 5.1 (0.4-9.8) | 0.5 (0-2.8) | 10.7 (5.2-19.9) | 3.1 (0-21.2) | 15.5(9.1-23.1) | 3.5 (0-21.7) |
| Sweet snack foods | 7.5 (3.6-14.6) | 11.8 (3.7-24.2) | 13.3 (7.8-22.7) | 26.4(13.7-43.5) | 15.1(8.1-23.8) | 29.9(15.9-49.8) |
| Savoury snack foods | 1.7 (0.4-5.3) | 0 (0-0.6) | 3.1 (1.2-6.5) | 0.3 (0-5.3) | 4.4 (1.9-9.4) | 4.6 (1.2-11.4) |
| Crispbreads and crackers | 5.4 (2.7-10.1) | 2.3 (0-5.0) | 5.7 (2.2-9.6) | 2.7 (0.4-6.8) | 5.7 (0.4-5.3) | 3.5 (0.4-10.0) |
| Nuts and seeds | 0.4 (0.1-1.1) | 0.1 (0-0.3) | 1.7 (0.2-5.2) | 0.8 (0-4.4) | 1.6 (0.4-5.3) | 1.1 (0-3.4) |
| Butter and margarine | 3.6 (1.5-5.1) | 1.9 (0.7-3.4) | 4.5 (1.9-6.4) | 3.0 (1.1-5.6) | 5.0 (2.6-8.5) | 3.5 (1.4-6.5) |
| Sugars, jams and honey | 0.5 (0.1-1.2) | 0.1 (0-1.9) | 1.4 (0.5-3.3) | 2.3 (0.2-10.3) | 1.2   (0.6-3.4) | 4.0 (0.3-11.9) |
