# Supplementary material for: Development and evaluation of a food frequency questionnaire for use among young children
Source: PLoS One. 2020 Mar 25;15(3):e0230669. doi: 10.1371/journal.pone.0230669 (PMC7094848; doi:10.1371/journal.pone.0230669)
Supplement: S1 File — (PDF) [file pone.0230669.s005.pdf]

59974

SECTION D: YOUR CHILD'S FOOD AND EATING

QD1 About how many serves of vegetables does your child usually eat per day? Do not include potatoes, hot chips or fried potato. (1 serve = ½ cup cooked vegetables or 1 cup salad vegetables) (Please shade *one* response only)

My child does not eat vegetables	Less than one serve/day	1 serve/day	2 serves or more/day
O 0	O 1	O 2	O 3

QD2 About how many serves of hot chips, French fries, wedges, or fried potatoes does your child usually eat per week? (1 serve = a small cup) (Please shade *one* response only)

My child does not eat chips	Less than one serve/week	1 serve/week	2 serves/week
O 0	O 1	O 2	O 3

QD3 About how many serves of potatoes does your child usually eat per week? Do not include chips, French fries, wedges or fried potatoes. (1 serve = 1 small potato) (Please shade *one* response only)

My child does not eat potatoes	Less than one serve/week	1 serve/week	2 serves/week
O 0	O 1	O 2	O 3

QD4 About how many serves of fruit does your child usually eat per day? Do NOT include fruit juice. (1 serve = 1 medium piece or 2 small pieces of fruit or 1 cup of diced pieces) (Please shade *one* response only)

My child does not eat fruit	Less than one serve/day	1 serve/day	2 serves or more/day
O 0	O 1	O 2	O 3

QD5 What types of bread does your child usually eat? (Please shade *any* that your child usually eats)

My child does not eat bread	High fibre white bread	White bread	Wholemeal bread	Rye bread	Multigrain bread	Other bread
O 0	O 1	O 2	O 3	O 4	O 5	O 6

QD6 What type of milk does your child usually drink? (Please shade *one* response only)

My child does not drink milk	Whole (full-cream/ regular)	Skim	Low/reduced fat	Soy	Other	Don't know
O 0	O 1	O 2	O 3	O 4	O 5	O 6

QD7 About how often is the meat your child eats trimmed of fat either before or after cooking? (Please shade *one* response only)

My child does not eat meat	Never	Rarely	Sometimes	Usually	Always
O 0	O 1	O 2	O 3	O 4	O 5

59974

QD8 Does your child take any of the following supplements? (Please shade *one* response only)

	Never	Less than once/week	1-3 times/ week	4-6 times/ week	Every day
Multivitamin and/or mineral	○ 1	○ 2	○ 3	○ 4	○ 5
Fish oils	○ 1	○ 2	○ 3	○ 4	○ 5
Iron (separate from the multivitamin/mineral above)	○ 1	○ 2	○ 3	○ 4	○ 5
Vitamin C (separate from the multivitamin/mineral above)	○ 1	○ 2	○ 3	○ 4	○ 5

The next questions ask how many times your child has eaten certain foods over the past month.

We are not asking how much of each food s/he ate, we are asking how often they ate each food in the past month. Please respond by shading the box that best matches your answer.

EXAMPLE ONLY

Question: How often did your child eat/drink this food/beverage *over the last month?*
(Please shade *one* response on each line)

If, for example, during each average week over the past month, your child:

- had cake once per week
- had bananas three times per week
- had toast for breakfast each morning and a sandwich for lunch on each weekday, this means that, on average, they have had bread about twice a day
- had Coco Pops three times per week and Weetbix four times

Then this is how you would complete the questions:

	Never or less than once a month	1-3 times a month	Once a week	2-4 times a week	5-6 times a week	Once a day	2-3 times a day	4-5 times a day	6 or more times a day
Cakes, muffins, scones, muesli bars	○ 1	○ 2	● 3	○ 4	○ 5	○ 6	○ 7	○ 8	○ 9
Banana	○ 1	○ 2	○ 3	● 4	○ 5	○ 6	○ 7	○ 8	○ 9
Bread	○ 1	○ 2	○ 3	○ 4	○ 5	○ 6	● 7	○ 8	○ 9
Breakfast cereal such as Weetbix, Vitabrits, Sultana bran	○ 1	○ 2	○ 3	● 4	○ 5	○ 6	○ 7	○ 8	○ 9
Breakfast cereal such as Nutrigrain, Rice Bubbles, Corn Flakes, Coco Pops	○ 1	○ 2	○ 3	● 4	○ 5	○ 6	○ 7	○ 8	○ 9

59974

QD9 In the past month, about how often has your child had the following? (Please shade *one* response on each line)

MILK & DAIRY FOODS	Never or less than once a month	1-3 times a month	Once a week	2-4 times a week	5-6 times a week	Once a day	2-3 times a day	4-5 times a day	6 or more times a day
Plain milk	○ 1	○ 2	○ 3	○ 4	○ 5	○ 6	○ 7	○ 8	○ 9
Infant formula, follow-on formula or toddler milk	○ 1	○ 2	○ 3	○ 4	○ 5	○ 6	○ 7	○ 8	○ 9
Flavoured milk drinks (e.g. milkshakes, smoothies, hot chocolate)	○ 1	○ 2	○ 3	○ 4	○ 5	○ 6	○ 7	○ 8	○ 9
Soy milk	○ 1	○ 2	○ 3	○ 4	○ 5	○ 6	○ 7	○ 8	○ 9
Yoghurt - plain or flavoured	○ 1	○ 2	○ 3	○ 4	○ 5	○ 6	○ 7	○ 8	○ 9
Cheese - all types	○ 1	○ 2	○ 3	○ 4	○ 5	○ 6	○ 7	○ 8	○ 9
Ice cream or custard	○ 1	○ 2	○ 3	○ 4	○ 5	○ 6	○ 7	○ 8	○ 9
Cream or sour cream	○ 1	○ 2	○ 3	○ 4	○ 5	○ 6	○ 7	○ 8	○ 9

BREAD & CEREAL FOODS	Never or less than once a month	1-3 times a month	Once a week	2-4 times a week	5-6 times a week	Once a day	2-3 times a day	4-5 times a day	6 or more times a day
Bread and bread rolls, English muffin, bagel or crumpet	○ 1	○ 2	○ 3	○ 4	○ 5	○ 6	○ 7	○ 8	○ 9
Breakfast cereal such as Nutrigrain, Rice Bubbles, Corn Flakes, Coco pops	○ 1	○ 2	○ 3	○ 4	○ 5	○ 6	○ 7	○ 8	○ 9
Breakfast cereal such as Weetbix, Vitabrits, Sultana bran	○ 1	○ 2	○ 3	○ 4	○ 5	○ 6	○ 7	○ 8	○ 9
Rice – white or brown	○ 1	○ 2	○ 3	○ 4	○ 5	○ 6	○ 7	○ 8	○ 9
Pasta or noodles – white or wholemeal	○ 1	○ 2	○ 3	○ 4	○ 5	○ 6	○ 7	○ 8	○ 9

59974

QD9cont. **In the past month, about how often has your child had the following?** (Please shade *one* response on each line)

**MEAT, FISH & EGGS**	Never or less than once a month	1-3 times a month	Once a week	2-4 times a week	5-6 times a week	Once a day	2-3 times a day	4-5 times a day	6 or more times a day
Beef or lamb (e.g. steak, roast)	○ 1	○ 2	○ 3	○ 4	○ 5	○ 6	○ 7	○ 8	○ 9
Dishes with beef or lamb (e.g. casserole, stir-fry)	○ 1	○ 2	○ 3	○ 4	○ 5	○ 6	○ 7	○ 8	○ 9
Ham, luncheon meat, bacon, salami	○ 1	○ 2	○ 3	○ 4	○ 5	○ 6	○ 7	○ 8	○ 9
Chicken – roast, steamed, BBQ	○ 1	○ 2	○ 3	○ 4	○ 5	○ 6	○ 7	○ 8	○ 9
Crumbed chicken (e.g. chicken nuggets, schnitzel)	○ 1	○ 2	○ 3	○ 4	○ 5	○ 6	○ 7	○ 8	○ 9
Pork – roast, chop or steak	○ 1	○ 2	○ 3	○ 4	○ 5	○ 6	○ 7	○ 8	○ 9
Dishes with pork, bacon or ham (e.g. casserole, stir-fry)	○ 1	○ 2	○ 3	○ 4	○ 5	○ 6	○ 7	○ 8	○ 9
Fish - fried, battered, crumbed (includes fish fingers)	○ 1	○ 2	○ 3	○ 4	○ 5	○ 6	○ 7	○ 8	○ 9
Fresh fish or seafood or canned fish (e.g. tuna, salmon)	○ 1	○ 2	○ 3	○ 4	○ 5	○ 6	○ 7	○ 8	○ 9
Dishes with fish or seafood (e.g. casserole, stir-fry)	○ 1	○ 2	○ 3	○ 4	○ 5	○ 6	○ 7	○ 8	○ 9
Sausages and Frankfurts (all types- boiled, grilled or fried)	○ 1	○ 2	○ 3	○ 4	○ 5	○ 6	○ 7	○ 8	○ 9
Eggs- boiled, scrambled	○ 1	○ 2	○ 3	○ 4	○ 5	○ 6	○ 7	○ 8	○ 9

**BEVERAGES**	Never or less than once a month	1-3 times a month	Once a week	2-4 times a week	5-6 times a week	Once a day	2-3 times a day	4-5 times a day	6 or more times a day
Plain water (e.g. tap water, bottled water, filtered water, unflavoured mineral water)	○ 1	○ 2	○ 3	○ 4	○ 5	○ 6	○ 7	○ 8	○ 9
Fruit juice (100% juice)	○ 1	○ 2	○ 3	○ 4	○ 5	○ 6	○ 7	○ 8	○ 9
Soft drink, cordial, sports drinks or fruit juice drinks	○ 1	○ 2	○ 3	○ 4	○ 5	○ 6	○ 7	○ 8	○ 9
Diet soft drinks or cordial	○ 1	○ 2	○ 3	○ 4	○ 5	○ 6	○ 7	○ 8	○ 9

59974

QD9cont. **In the past month, about how often has your child had the following?** (Please shade *one* response on each line)

MISCELLANEOUS FOODS

	Never or less than once a month	1-3 times a month	Once a week	2-4 times a week	5-6 times a week	Once a day	2-3 times a day	4-5 times a day	6 or more times a day
Cakes, muffins, scones, muesli bars	O 1	O 2	O 3	O 4	O 5	O 6	O 7	O 8	O 9
Sweet biscuits - all types	O 1	O 2	O 3	O 4	O 5	O 6	O 7	O 8	O 9
Chocolate and sweets/lollies	O 1	O 2	O 3	O 4	O 5	O 6	O 7	O 8	O 9
Crisps, corn chips, Twisties	O 1	O 2	O 3	O 4	O 5	O 6	O 7	O 8	O 9
Crackers and crispbreads (e.g. rice cakes)	O 1	O 2	O 3	O 4	O 5	O 6	O 7	O 8	O 9
Savoury biscuits (e.g. Jatz, Shapes)	O 1	O 2	O 3	O 4	O 5	O 6	O 7	O 8	O 9
Savoury pies and pastries (e.g. sausage roll, meat pies, pastie)	O 1	O 2	O 3	O 4	O 5	O 6	O 7	O 8	O 9
Hamburgers (e.g. McDonalds)	O 1	O 2	O 3	O 4	O 5	O 6	O 7	O 8	O 9
Pizza	O 1	O 2	O 3	O 4	O 5	O 6	O 7	O 8	O 9
Peanuts, peanut butter, other nuts seeds or tahini	O 1	O 2	O 3	O 4	O 5	O 6	O 7	O 8	O 9
Sugar, jam, honey, syrups	O 1	O 2	O 3	O 4	O 5	O 6	O 7	O 8	O 9
Butter or dairy spreads on bread or cooked vegetables	O 1	O 2	O 3	O 4	O 5	O 6	O 7	O 8	O 9
Margarine on bread or cooked vegetables	O 1	O 2	O 3	O 4	O 5	O 6	O 7	O 8	O 9

VEGETABLES (including fresh, frozen and tinned)

	Never or less than once a month	1-3 times a month	Once a week	2-4 times a week	5-6 times a week	Once a day	2-3 times a day	4-5 times a day	6 or more times a day
Hot chips, potato wedges	O 1	O 2	O 3	O 4	O 5	O 6	O 7	O 8	O 9
Potato - Boiled, baked	O 1	O 2	O 3	O 4	O 5	O 6	O 7	O 8	O 9
Carrot	O 1	O 2	O 3	O 4	O 5	O 6	O 7	O 8	O 9
Tomato	O 1	O 2	O 3	O 4	O 5	O 6	O 7	O 8	O 9
Broccoli	O 1	O 2	O 3	O 4	O 5	O 6	O 7	O 8	O 9

59974

QD9cont. **In the past month, about how often has your child had the following?** (Please shade *one* response on each line)

VEGETABLES cont.

	Never or less than once a month	1-3 times a month	Once a week	2-4 times a week	5-6 times a week	Once a day	2-3 times a day	4-5 times a day	6 or more times a day
Peas	O 1	O 2	O 3	O 4	O 5	O 6	O 7	O 8	O 9
Cauliflower	O 1	O 2	O 3	O 4	O 5	O 6	O 7	O 8	O 9
Cucumber	O 1	O 2	O 3	O 4	O 5	O 6	O 7	O 8	O 9
Capsicum	O 1	O 2	O 3	O 4	O 5	O 6	O 7	O 8	O 9
Onion	O 1	O 2	O 3	O 4	O 5	O 6	O 7	O 8	O 9
Corn	O 1	O 2	O 3	O 4	O 5	O 6	O 7	O 8	O 9
Pumpkin	O 1	O 2	O 3	O 4	O 5	O 6	O 7	O 8	O 9
Zucchini	O 1	O 2	O 3	O 4	O 5	O 6	O 7	O 8	O 9
Mushroom	O 1	O 2	O 3	O 4	O 5	O 6	O 7	O 8	O 9

FRUITS (including fresh, frozen and tinned)

	Never or less than once a month	1-3 times a month	Once a week	2-4 times a week	5-6 times a week	Once a day	2-3 times a day	4-5 times a day	6 or more times a day
Apple and pear	O 1	O 2	O 3	O 4	O 5	O 6	O 7	O 8	O 9
Orange, mandarin and other citrus fruit	O 1	O 2	O 3	O 4	O 5	O 6	O 7	O 8	O 9
Banana	O 1	O 2	O 3	O 4	O 5	O 6	O 7	O 8	O 9
Grapes	O 1	O 2	O 3	O 4	O 5	O 6	O 7	O 8	O 9
Strawberries	O 1	O 2	O 3	O 4	O 5	O 6	O 7	O 8	O 9
Watermelon	O 1	O 2	O 3	O 4	O 5	O 6	O 7	O 8	O 9
Kiwi fruit	O 1	O 2	O 3	O 4	O 5	O 6	O 7	O 8	O 9
Peach, apricot, nectarine, plum	O 1	O 2	O 3	O 4	O 5	O 6	O 7	O 8	O 9
Pineapple	O 1	O 2	O 3	O 4	O 5	O 6	O 7	O 8	O 9
Rockmelon	O 1	O 2	O 3	O 4	O 5	O 6	O 7	O 8	O 9
Dried fruit (e.g. sultanas, apricots)	O 1	O 2	O 3	O 4	O 5	O 6	O 7	O 8	O 9
Fruit - canned in syrup or cooked with sugar	O 1	O 2	O 3	O 4	O 5	O 6	O 7	O 8	O 9
