# Supplementary material for: Development and evaluation of a food frequency questionnaire for use among young children
Source: PLoS One. 2020 Mar 25;15(3):e0230669. doi: 10.1371/journal.pone.0230669 (PMC7094848; doi:10.1371/journal.pone.0230669)
Supplement: Attachment — Submitted filename: FFQ PLOS Reviewer comments_2019 august.docx [file pone.0230669.s006.docx]

**PONE-D-17-29484**

**DEVELOPMENT AND EVALUATION OF A FOOD FREQUENCY QUESTIONNAIRE FOR USE AMONG YOUNG CHILDREN**

**Reviewer comments**

In your revised **cover letter**, please address the following prompts:

a) If there are ethical or legal restrictions on sharing a de-identified data set, please explain them in detail (e.g., data contain potentially identifying or sensitive patient information) and who has imposed them (e.g., an ethics committee). Please also provide contact information for a data access committee, ethics committee, or other institutional body to which data requests may be sent.

b) If there are no restrictions, please upload the minimal anonymized data set necessary to replicate your study findings as either Supporting Information files or to a stable, public repository and provide us with the relevant URLs, DOIs, or accession numbers. Please see <http://www.bmj.com/content/340/bmj.c181.long> for guidelines on how to de-identify and prepare clinical data for publication. For a list of acceptable repositories, please see <http://journals.plos.org/plosone/s/data-availability#loc-recommended-repositories>.

We will update your Data Availability statement on your behalf to reflect the information you provide.

**Response**

We have now addressed the data sharing in the cover letter. Data cannot be shared publicly because of ethical consideration due to potential identifying information. Data are available from the Deakin University Institutional Data Access (contact via karen.campbell@deakin.edu.au) for researchers who meet the criteria for access to confidential data.

**ADDITIONAL EDITOR COMMENTS:**

A thoughtfully designed study. A well-written manuscript on a methodology that seems rigorous and most of the results are appropriately presented. You should explain whether selection bias or systematic bias has occurred. As the attenuation coefficient is a commonly used measure of relative validity it would be important to report these also. Since the tables are too large, converting some of the tables to figures would be an option. P-values are irrelevant, magnitude of the kappa statistic and correlations should be sufficient.

To improve your study, interesting covariates that were collected and only mentioned only could be used for a regression analysis. Finally in the conclusions it is relevant to state for what purposes you would recommend this instrument.

**REVIEWER #1**

The authors describe the development and evaluation of a food frequency questionnaire for use in Australian infants and children. Overall, the instrument appears to have been developed using rigorous methodology, the results appropriately presented, and the manuscript well-written. However, the manuscript may benefit from minor changes in the approaches used for evaluation and a more nuanced interpretation of the findings. My specific comments are provided below.

**Abstract:**

1.) Minor – the term “three-day 24 hour recalls” used throughout is confusing as written. “Three non-consecutive 24-hour recalls” is likely preferable to limit potential confusion regarding the time frame covered by the recall.

**Response**

The terminology has been changed in the abstract and throughout the paper to “three non-consecutive 24-hour recalls” in L16.

2.) Minor – some mention of the time frame covered by FFQ may help orient readers (i.e. past month, year, etc.)

**Response**

The timeframe “over the past months” has been added to the abstract—background/objective section in L15.

3.) Minor – the abstract may be improved by more clearly specifying that the same children were examined over time.

**Response**

The abstract has been edited to reflect that the same children were followed over time: **“**Food and nutrient intakes from a 68-item FFQ were compared with three non-consecutive 24 hour recalls in a follow-up cohort of children aged 1.5, 3.5 and 5.0 years old” in L18.

4.) Minor – the authors state at lines 25 and 26 that the correlations were higher at older ages. Was this formally tested?

**Response**

We have now tested the equality of mean correlation coefficient between age groups and found the mean correlation coefficient were not significantly different. The following changes have now been made. The wording “correlations tended to be higher at older ages” has now been removed in the abstract. The sentence: “Equality of average correlation coefficients between each time point was tested by “CORTESTI” command” is added to the method section in L171-172.

“Comparison of equality of average Pearson’s r between each time points revealed no significant difference” and “No significant difference between Spearman’s r between time points were found” are added to the results section in L201 and in L236, respectively.

5.) Major – the conclusion does not appear to provide much useful information with respect to how one should “view” this instrument or inform the reader on its use. For example, are the authors recommending that this instrument be used to measure dietary intake in infants and children? If so, for what groups or for what specific foods/nutrients. Are there some foods/nutrients for which the authors would not recommend the use of this instrument (i.e. kappa or rho too low to be informative)? In addition, for what purposes would the authors recommend using this instrument?

To compare means intakes across groups and/or for use in diet-health outcome research? How might the correlations (and attenuation factors) for specific foods and nutrients be expected to impact regression parameters and sample size requirements accounting for the expected degree of error? Is it expected to be the same for infants and older children? More detailed discussion (summarized here but provided in detail in the discussion) would greatly contribute to the usefulness of this research.

**Response**

Thank you for your valuable suggestions. We have now incorporated this in the manuscript. The conclusion of the abstract now reads: At all three time points, the FFQ demonstrated fair agreement for most nutrient and food intakes when compared with 24-hour recalls. This FFQ is a valid and reliable tool for assessing total diet and ranking individuals according to nutrient and food intakes among young children. The conclusion of the paper now reads: This study evaluated an FFQ to assess the food group and nutrient intakes in young children aged 1.5 to 5 years. At all three time points, and consistent with other studies, the FFQ produced higher estimates of intake for the majority of nutrients and food groups that were examined when compared with estimates derived from 24-hour recalls, although it demonstrated fair agreement for most nutrient and food intakes. Further assessment of portion sizes and validation testing in different population groups is warranted. However, this FFQ is a valid and reliable tool for assessing total diet and ranking individuals according to nutrient and food intakes. The tool is suitable for monitoring dietary intake among young children and has application for assessing diet and disease relationships in large population-based studies and dietary interventions among young children under the five years of age.

Introduction:

6.) Minor – at line 47 the authors state advantages of the FFQ to other self-report dietary instruments is the lower cost; however, there are now self-administered 24-hours and FFQ available in the U.S. for such studies. Perhaps some elaboration on this and whether such resources are available to researches in Australia might help clarify this statement.

**Response**

This sentence “resulting in lower research costs” has been removed to acknowledge the availability of self-administered online tools although their use in Australia is recent, and primarily available for use in adults.

7.) Minor – (line 48) FFQ are generally not well-suited to assess the impact of an intervention given they inherently are designed to query generic memory regarding patterns of usual food consumption. Thus, the information obtained from such an instrument may be expected to be more prone to recall bias that an instrument attempting to query foods actually consumed (i.e. 24 hour recall). Of course both are limited in this regard and the inclusion of an objective marker of dietary intake or status be included when attempting to assess the impact of an intervention whenever possible.

**Response**

Nutrition intervention has now been replaced with large scale epidemiological studies and the sentence now reads “FFQs can provide useful measures of dietary intake suitable for large scale epidemiological studies and investigating diet and diseases relationships” in L50.

**Methods:**

8.) Major – less than half of the study subjects were included in the instrument evaluation. Can the authors rule out that that this did not result in a selection bias that might have impacted the results and their generalizability? If not, some statement to this effect may be warranted in the limitations. A rigorous comparison of the included and excluded subjects on relevant measures would provide some indication.

**Response**

Thanks for your advice. The comparison of those included versus excluded from the analysis is now provided as a supplementary table. The following text is added to the manuscript under “Analytical Sample” in L144-147. Comparison of children who included versus excluded from the analysis is presented in S1 Table. Children who included in the analysis were slightly younger than those excluded from the analysis with mean difference ranging from 0.1 to 0.3 years. However, no significant difference was found in regards to sex, maternal education, and BMI z-score.

9.) Major – the design allow for some subjects to have scheduled recalls and for others to use a 24-hor recall + food diary model for dietary intake assessment. Did the authors examine whether this may have results in any systematic bias in terms of the performance of the instrument? What was done here may reflect the best possible approach to assessing dietary intake for these children, but there should be some sensitivity analyses conducted to ensure the performance in generally similar across approaches.

**Response**

Thank you for your great point. In our sample, 4% of total interviews were pre-scheduled and used the food diary model. We have now added this as a limitation to our study in L330-333: “Pre-scheduled interview plus food diary was used to capture dietary intake from children caring by more than one carer (4%). However, the impact of this alternative approach on the results is likely to be small given the small percentage.”

10.) Minor – It is well understood that the number of item included on a FFQ will dictate absolute amounts of energy, food, and nutrients. The FFQ does not appear to have been designed to align with absolute intakes as measure by a reference instrument (for example 24 hour recalls as used here), but rather only explaining variation in key nutrients and indictors of diet quality were used in the development. Might this explain the higher mean values for the FFQ across most groups (i.e. the purpose when designing the instrument looks to be focused on ranking/discriminating subjects on intake rather than accurately assessing mean intake)?

**Response**

Thanks for your comments. We do agree the design of FFQ is to allow assessment of total diet and ranking of individuals according to intake rather than capture actual intake. The overestimation of FFQ in comparison to 24 hr recalls is likely due to the use of portion size from the Australian National Children’s Survey. Unable to measure portion size along with the FFQ has been noted as a limitation to our study. Also, compared to 24 hr recalls, FFQ captures usual intake which may in part explain the discrepancies in intakes. Overestimation of intake by FFQ compared with dietary recalls is consistently reported by other studies. These discussions have already been outlined in our study.

11.) Major – the authors used repeated 24-hours as the reference instrument, but did not consider correcting/deattenuating correlation coefficients when assessing performance. Three 24-hour recalls are known to be subject to a high-degree of intra-individual variation that would be expected to downwardly biased correlations estimating true usual intake between instruments (i.e. unfairly penalizing the comparison being made). In addition, the attenuation coefficient is a commonly used measure of relative validity and would be important to report here.

**Response**

The de-attenuated correlation coefficients are now calculated and presented accordingly in the method, result and tables. The following text is added to the method section: “To account for random within person variation among three 24-hour recalls, the energy-adjusted correlation coefficients were de-attenuated based on the formula: $R=r\sqrt{1+ʎ/3}$, in which the attenuation factor ($ʎ$ ) is the ratio of within- and between-person variances obtained from the three recalls (Willett W. Nutritional epidemiology. New York: Oxford University Press; 2012)” in L162-165. After correcting for within person variation, the de-attenuated correlation coefficient for all nutrients and foods improved. The following text has been added to the manuscript: “Accounting for within person variation, the Pearson correlation at all time points for all nutrients improved, and the average de-attenuated Pearson’s R was 0.33, 0.38 and 0.37 at 1.5, 3.5 and 5 years, respectively” in results L202-203.”Spearman correlation for all food intakes at all time points improved after correcting for within person variation. The average de-attenuated Spearman’s R for food intakes was 0.36, 0.39, and 0.39 at 1.5, 3.5, and 5.0 years, respectively” in results L237-238.

“Correcting for within person variation among three 24-hour recalls improved the correlations” in discussion L262-L263.

12.) Minor – more information is needed at line 145 on how the groups were collapsed and whether this may have contributed to mean differences between instruments.

**Response**

Total 68 FFQ items were collapsed into 26 food groups by grouping similar foods. Detailed categorisation of 26 food groups is provided in S2 Table in L154.

13.) Minor – a statement is required as top why these specific foods and nutrients were considered for the evaluation.

**Response**

For ease of comparison, similar food items within FFQ were grouped together and resulted in a total of 26 food groups. For nutrient intake (in L155-157), key nutrients assessed are those relevant to obesity (energy, fat, saturated fat, sugars) and key indicators of diet quality among young children (protein, fibre, vitamin C and folate as markers of fruit and vegetable intake, and iron and calcium).

14.) Major – the 24-hour recalls and the FFQ appear to be assessing dietary intake occurring over different time periods? At line 167 in the results it is stated that the mean number of days between instruments was around 6 days. It is my understanding that the FFQ was querying dietary intake occurring over the past month. Perhaps a figure (supplemental) may be informative in making the assessment period clear to readers. If the recalls and FFQ are assessing intake occurring over different time periods it becomes challenging/impossible to properly disentangle true differences in the foods/nutrients consumed and artificial variation attributable to differences in the instruments. Further discussion of this should be provided and listed as a study limitation if this is fact the case.

**Response:**

A figure below outlines the assessment period between three 24-hour recalls and FFQ is now provided as S1 Fig in L183.

Number of days between 24-hour recall and FFQ: <7 days

24 hr recall 2

Weekday

24 hr recall 3

Weekend day

FFQ

24 hr recall 1

Weekday

This has now also been noted in limitation: “Another limitation of the study is the gap between measurement of 24-hour recalls and FFQ. Although the number of days between two measures were less than a week, it could potentially affect the variation in intake and results in difference between two instruments “ in L327-329.

**Discussion:**

15.) Minor – at line 279 it is stated that the use of multiple 24-hour recalls is a strength of this study. Multiple studies assessing the performance of the 24-hour recall and FFQ to recovery biomarkers have demonstrated correlated errors in these instruments questioning the accuracy of the 24-hour recall as the reference instrument of such studies. While the recall is commonly used as the reference instrument when evaluating the FFQ, it seems a hard sell to consider its use as the reference instrument a strength of the study. Rather a strength would have been the inclusion of objective measures of intake (acknowledging the challenges).

**Response**

Addition of reference biomarkers would have been desirable. Unfortunately, our study have no funding available for inclusion in this study. We have now removed the use of 24 hour recall as a reference as a strength and added the inclusion of objective measures of intakes as a strength to our study. We have edited this section of the discussion and acknowledged this in the discussion in L301.

16.) Minor – the claim at line 284 that the 24-hour recall should be used as the reference instrument because it is cited to have shown the most accurate estimate of total energy intake seems dubious. Numerous studies have shown that the correlation between the 24-hour recall and FFQ with doubly labeled water are too low to be useful. Some assessment of the magnitude of how accurate the recall is for this purpose should be provided so as not to mislead readers.

**Response**

Thank you for your thoughts. This sentence has now been removed.

**REVIEWER #2**

This is a thoughtfully designed study to determine the accuracy of a children's food frequency questionnaire by using kappa statistics to establish agreement with carer recall. The methodology seems sound, and the authors clearly know the literature on dietary questionnaires. There are a few items that could improve the paper:

1. The tables are so voluminous that they are really not interesting to the reader. They need to be cut dramatically. I would suggest converting some of the tables to figures.

**Response**

We have now removed the median intakes of foods estimated from FFQ and 24 hour recalls from the Table 4 to the S3 Table.

2. P-values have no meaning in an exploratory study with hundreds of "hypothesis tests". But you are not really testing hypotheses. The magnitude of the kappa statistic and correlations should be sufficient to indicate what's going on. I would delete all the asterisks in the tables. The hypothesis that kappa=0 is highly uninteresting and your type I error rate is so high that reporting p-values is meaningless.

**Response**

P-Values have now been deleted from the tables.

3. The interesting covariates that were collected are mentioned only tangentially. How about a regression analysis to see which covariates are predictive of more accurate agreement? You obviously are interested, for instance, in education level of the carers.

**Response**

The covariates were provided in order to describe the target population and understand the nature of the participants in the study, and for comparison. While this analysis would be an interesting addition to the paper, given the large amount of data in the paper already as noted by this reviewer, we have not included this analysis as it is beyond the scope of this evaluation.

**Journal Requirements:**

1. When submitting your revision, we need you to address these additional requirements.

Please ensure that your manuscript meets PLOS ONE's style requirements, including those for file naming. The PLOS ONE style templates can be found at <http://www.journals.plos.org/plosone/s/file?id=wjVg/PLOSOne_formatting_sample_main_body.pdf> and <http://www.journals.plos.org/plosone/s/file?id=ba62/PLOSOne_formatting_sample_title_authors_affiliations.pdf>

**Response:**

We have ensured that our manuscript meets all the style requirements.

2. Please state in your methods section whether you obtained consent from parents or guardians of the minors included in the study or whether the research ethics committee or IRB approved the lack of parent or guardian consent.

**Response**

We have amended the manuscript to clarify that parental consent was obtained in L74.

3. Please include a copy of the survey questions or questionnaire used in the study, in both the original language and English, as Supporting Information, or include a citation if it has been published previously."

**Response**

A copy of the questionnaire has now been provided as S1 file in L115.

4. We note that you have indicated that data from this study are available upon request. PLOS only allows data to be available upon request if there are legal or ethical restrictions on sharing data publicly. For information on unacceptable data access restrictions, please see <http://journals.plos.org/plosone/s/data-availability#loc-unacceptable-data-access-restrictions>.

**Response**

We have now addressed the data sharing in the cover letter. Data cannot be shared publicly because of ethical consideration due to potential identifying information. Data are available from the Deakin University Institutional Data Access (contact via karen.campbell@deakin.edu.au) for researchers who meet the criteria for access to confidential data.
