# Supplementary material for: Development and evaluation of a food frequency questionnaire for use among young children
Source: PLoS One. 2020 Mar 25;15(3):e0230669. doi: 10.1371/journal.pone.0230669 (PMC7094848; doi:10.1371/journal.pone.0230669)
Supplement: Attachment — Submitted filename: PLOS ONE FFQ Feb 2020 reviewers comments_revised.docx [file pone.0230669.s008.docx]

1) Per the Willett textbook quoted, Spearman correlation should be used for FFQ data, whether the data are normally distributed or not, as the FFQ aims to rank subjects within a cohort rather than to provide point estimates of usual intake.

Reply: Thank you for your advice. We agree that FFQ aims to rank subjects within a cohort rather than to provide point estimate of usual intake, and that Spearman correlation can indeed be used. As outlined below, however, most studies used Pearson correlation to compare nutrient intakes, and therefore we used Pearson correlation for consistency and comparison with other studies. Moreover, generally for two normally distributed variables, the results from the Pearson and Spearman values will be quite similar when the relationship is linear (usually reasonably true in the case of validation studies (Reference: Willett WC. Nutritional Epidemiology. New York: Oxford University Press; 2012. Chapter 6. DOI 10.1093/acprof:oso/9780199754038.003.0006, page 68 out of 91)

We carefully considered the type of validation test in our study and followed the recommendation from the Willet textbook and Margetts textbook to use Pearson Correlation to compare nutrient intakes between FFQ and the reference method (i.e. 24hr recall measures in our study) when data are normally distributed – and to use Spearman correlation to compared skewed food intakes (rational provided below). In both Chapter 6 of Willet’s textbook and Chapter 8 of the Margetts’s textbook, all examples on validity of FFQ nutrient intake against measures from the reference methods utilized Pearson Correlation.

The Margetts textbook notes: “*The most common method of assessing the validity of questionnaires is to test the agreement in ranking of subjects between questionnaire and standard.* *Consistency of ranking is usually measured using the Pearson product−moment correlation coefficient (often on log transformed data to improve approximation to the normal distribution), although other correlation coefficients (Spearman’s, intra-class) are also used*.” (Reference: Margetts BM, Nelson M. Design Concepts in Nutritional Epidemiology. Oxford University Press; 1997. DOI:10.1093/acprof:oso/9780192627391.003.0008, Chapter 8. (8.6.2). Ranking and regression, page 24 out of 43).

Further support for the use of Pearson correlation in FFQ validation studies has also been found in Lombard et al., who reviewed the range of statistical tests used in the validation of quantitative FFQ and indicated that Pearson Correlation is one of the main tests used in FFQ validation studies to assess the correlation coefficient. (References: Lombard MJ, Steyn NP, et al. Application and interpretation of multiple statistical tests to evaluate validity of dietary intake assessment methods. Nutrition Journal. 2015: 14, 40)

In considering skewed data, Willett notes: *“Because dietary variables are usually skewed toward higher values, transformations (such as log) to increase normality should be considered before computing correlation coefficients. This has the advantage of reducing the influence of extreme values and of creating a correlation coefficient that can be interpreted in the form of a contingency table. Alternatively, nonparametric correlation coefficients (e.g., Spearman) can be used when one or both variables are not normally distributed.*” We provide this quote to justify our use of spearman correlation to compare skewed food intakes. (Reference: Willett WC. Nutritional Epidemiology. New York: Oxford University Press; 2012. Chapter 6. DOI 10.1093/acprof:oso/9780199754038.003.0006, page 71 out of 91).

2) Given the aim of FFQ to rank subjects rather than provide point estimates of usual intake, the energy intake calculations are at best gross estimates. The use of national median portion sizes also adds significant error to these estimates. These limitations should be more clearly emphasized in discussing that approach and in interpreting the results.

Reply: Thank you for your comments. Please see edits below to further highlight the limitations and assist readers with use of our tool in future studies.

When utilization this FFQ tool in future studies researchers should be aware that the FFQ aims to rank subjects rather than provide true estimates of usual intakes. Special consideration on measurement error and dietary misreporting for analysing and interpreting self-reported data including FFQ in dietary surveillance and nutritional epidemiology is needed (Subar et al). Now added as Line 362-365

To ease subject burden and ensure generalisability of the FFQ at the population level, we did not ask respondents to estimate portion size in the FFQ and used reference portion sizes from a national survey to estimate intakes. This may have introduced significant error and resulted in the higher absolute intake of nutrient and foods from the FFQ. (in Line 383-387)

Subar A. et al. Addressing Current Criticism Regarding the Value of Self-Report Dietary Data.J Nutr. 2015 Dec;145(12):2639-45. https://www.ncbi.nlm.nih.gov/pubmed/26468491

3) The authors’ response:

“Although our FFQ validation is nested in an early childhood obesity prevention study, the children involved in our study were not overweight or obese at the start of the trial. We made no exclusion of children in terms of their birth weight or nutritional status. Therefore, the utility of our FFQ is not limited to programs seeking to prevent overweight and obesity only and it has validity in assessing dietary intake among general Australian children….” Is very convincing and should be included in the Discussion, as many readers will have the same question.

Reply: Thank you for your suggestion. These sentences have now been added to the discussion in line 357-361.
